# The Clinical Utility of ABO and RHD Systems as Potential Indicators of Health Status, a Preliminary Study in Greek Population

**DOI:** 10.3390/clinpract12030045

**Published:** 2022-06-07

**Authors:** Evgenia Lymperaki, Evangelia Stalika, George Tzavelas, Efthymia Tormpantoni, Diana Samara, Eleni Vagdatli, Ioannis Tsamesidis

**Affiliations:** 1 Department of Biomedical Sciences, International Hellenic University, 57001 Thessaloniki, Greece; evlimper@gmail.com; 2 Lab of Computing and Medical Informatics, Medical School, Aristotle University of Thessaloniki, 54124 Thessaloniki, Greece; evangelia.stalika@gmail.com; 3 Department of Statistics and Insurance Science, University of Piraeus, 18534 Piraeus, Greece; tzafor@unipi.gr; 4 Blood Bank Section, Naousa General Hospital, 59200 Naousa, Greece; torbaef@yahoo.gr (E.T.); dianasamara7@gmail.com (D.S.); 5 Laboratory of Biopathology, Hippokratio General Hospital, 54642 Thessaloniki, Greece; vagdatli@mls.teithe.gr; 6 Faculty of Health Sciences, School of Dentistry, Aristotle University of Thessaloniki, 54124 Thessaloniki, Greece

**Keywords:** ABO blood system, Rhesus type, health biomarkers, lipid profile, atheromatic index, total antioxidant status

## Abstract

Objective: The objective of this study is to further highlight the differences between different ABO blood groups and Rhesus types with health biomarkers. Methods: In total 150 active healthy blood donors participated in our study comprising of 80 males from 19–61 years and 70 females aged from 21 to 64. Participants carrying blood group A were 55 individuals, blood group B 32, blood group O 51, and blood group AB 12, RHD+ 132, and RHD- 18. All the volunteer regular blood donors were selected recognizing them as a healthy population excluding drug and supplements intake. Their blood samples were analyzed just before blood donation for biochemical, hematological, and antioxidant markers. Statistical computations were performed using the SPSS tool, specifically, the one-way ANOVA test, Chi-square statistics, and logistic regression were used as statistical models. Results: O blood donors presented better iron absorption and the worst lipid profile. Indeed, a significant trend of high atheromatic index values revealed an increased risk for hyperlipidemia, in contrast with blood group A presenting a better lipid profile with lower atheromatic index values. There was also a gender related association for blood group A compared with O that was further highlighted using binary logistic regression. Conclusion: In this study, a significant difference was observed among the ABO blood groups in several of the examined biochemical and hematological biomarkers. O blood group appeared different behavior in comparison to all the tested blood groups and furthermore the RHD-group presented a better lipid profile in comparison to the RHD+ group. In order to obtain a more comprehensive view of the correlation between the ABO blood group and biochemical markers, further studies are required.

## 1. Introduction

The ABO system was discovered in the year of 1901 and afterward, an association between the ABO blood system and diseases such as malaria, hemolytic disorders, cancer, and life expectancy [1,2] was suggested. The antigens of ABO blood groups are highly expressed on red blood cells’ surfaces, such as on the surface of other human cells including vascular endothelium and platelets [3,4]. The different antigens consist of different carbohydrate determinants. This antigen system seems to be associated with different diseases [5,6]. The ABO blood group system categorizes the population into four blood groups: Individuals with blood group O creates antibodies to A and B antigens s, while blood group A and B have the A and B antigens, respectively, and the AB blood group do not make antibodies to others because they include both A and B antigens. A number of studies analyzed the influence of ABO Blood groups as a risk factor in cardiovascular disease (CVD). Increased blood lipid and inflammatory biomarkers, which are well known risk factors for CVD, are found in non O blood groups [7,8,9,10,11]. However, this association has not been confirmed maybe because of the small sample size of the studies [12]. The epidemiology of cardiovascular disease differs according to racial and ethnic groups. Although the African population have significantly higher von Willebrand Factor (VWF) levels than Caucasians and especially Africans with blood group O. In a study, AB blood group had a higher stroke risk in a large study among Africans–Americans, and they determined a non significant association between race ABO system and stroke [13]. Several studies reported that non O blood groups have more risk for thrombolytic disease, and O blood groups have the risk for hemorrhagic disorders. This observation is in agreement with the study about the association between the ABO blood system and levels of Factor VIIIc (FVIII) and von Willebrand factor (VWF) [14,15,16]. There were a few studies with a small population about the relationship between ABO blood groups and diabetes mellitus and showed a higher risk of diabetes in white European women with blood type B [9,17]. A clinical study in 2010 among Scandinavian blood donors followed for 35 years confirmed that blood group A is probably associated with gastric cancer [18]. Other studies showed a relationship between developing skin, ovarian, and lung cancer with the ABO system [19,20,21]. An association between the ABO blood group system and infectious diseases such as malaria, *H. pylori*, and cholera has been suspected mainly because of the absence or not of the A and B antigens. These antigens can be used as receptors, so the blood group O individuals seem to be protected [22,23,24] from the above diseases. Rh blood group is the second blood system that is more studied. The structure and function of the polypeptides associated with the Rhesus antigen could explain the role of those antigens [25]. The most common diseases associated with Rhesus are hemolytic anemia in newborns and chronic and autoimmune anemia [26]. Moreover, a couple of studies related to the ABO phenotype present links between genetically determined human ABO blood groups with increased risk of various infectious diseases [27]. The molecular level of ABO blood groups and their association with various diseases should be evaluated, too [28,29].

It is of great interest to point out the correlation of the RhD system with diseases as well. RhD protein is a component of NH(3) or CO(2) pump whose physiological role is unknown and is known to be able to cause hemolytic anemia, jaundice, and also liver and heart problems in newborns who are incompatible in their rhesus type with mothers rhesus type. Moreover, the absence of Rhesus protein is associated with allergic problems, digestive, heart, hematological, immunity, mental health, and neurological problems and they differ in their tolerance to certain biological factors, including Toxoplasma infection, aging, and fatigue.

The target of our study is to investigate if the ABO blood group, Rhesus types, and a combination of those, present a possible risk factor for the health benefits of the hematological (hematocrit and hemoglobin) and biochemical levels (serum iron and ferritin, total cholesterol, triglyceride, SGOT, SGPT, total antioxidant status) of regular blood donors. Consequently, we aimed to evaluate the role of ABO and RhD blood types as risk factors in pathological conditions (cardiovascular diseases, etc.).

## 2. Material and Methods

### 2.1. Study Design

**Figure 1:**
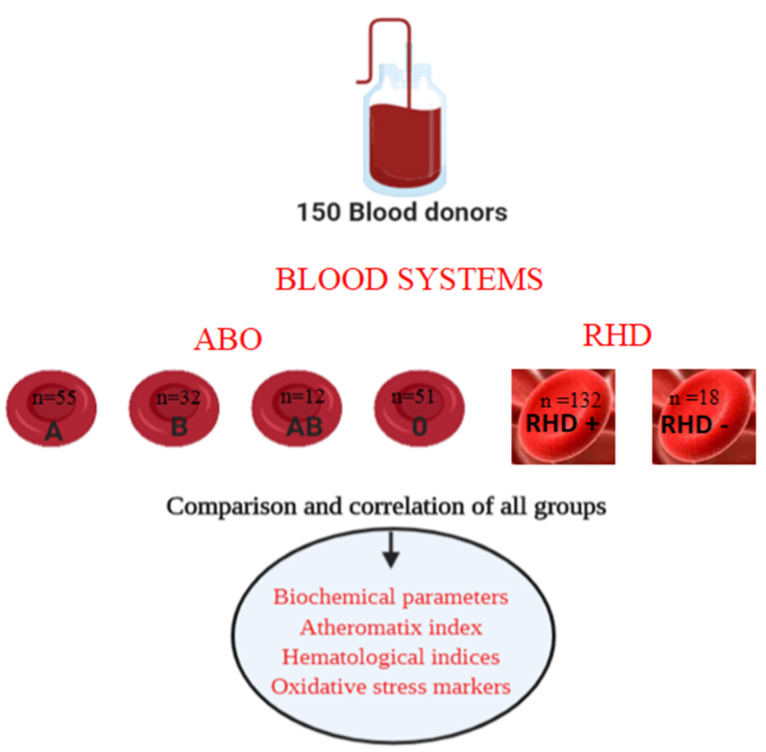
Flow diagram of the study. Number (*n*) of participants included in the study in regard to blood group classification.

A pilot study was carried out at the Blood Bank of Naousa Hospital Greece from January 2019 to December 2019, (Figure 1). The regular blood donors were comprised of 80 males aged 19–61 years, and 70 females aged 21–64 years were examined for biochemical and hematological markers in the blood sample which was taken just before blood donation.

The sample size was calculated using a single-population proportion for the outcome variable and a double-population proportion for factors. The final sample size was 150. All participants provided written informed consent before the study and were asked to fill out a short questionnaire about sociodemographic characteristics such as age, sex, body mass index (BMI), smoking habits, and residence of blood donors. All the volunteer regular blood donors (rBDs) were selected recognizing them as a healthy population, excluding drug supplement intake and females with menstruation and recent childbirth.

### 2.2. Blood Collection

Just before blood donation, 10 mL of blood samples were collected in two tubes for biochemical and hematological analysis (without anticoagulant and with K_3_ETDA, respectively). For antioxidant status analysis, the blood was centrifuged, and the serum was stored at −80 °C.

### 2.3. Blood Analysis

Cholesterol, Triglycerides, HDL- and LDL-cholesterol, Fe, Ferritin, uric acid, glucose, aspartate aminotransferase (AST), and alanine aminotransferase (ALT) levels were measured using a biochemical analyzer ROCHE in the laboratory of the General Hospital of Naousa. Serum levels of Total Antioxidant Status (TAS) was measured photometrically using the TAS assay kit of Cayman in the laboratory of the International Hellenic University.

Hematocrit and Hemoglobin (Hb) was also measured in Abbott Ruby cell_dyn, Roche Integra 400 plus in the laboratory of the General Hospital of Naousa.

### 2.4. ABO Blood & Rhesus System Detection

A, B, O, AB, and RhD groups were obtained for all participants from the Blood Bank in the General Hospital in Naousa using a column agglutination anti-A, anti-B anti-D Ortho Biovue clinical diagnostics serological system [30].

### 2.5. Ethical Statement

The study was conducted in accordance with Good Clinical Practice guidelines and the Declaration of Helsinki. Ethical approval to perform the present study was obtained from the Ethical committee of the General Hospital of Naousa. The confidentiality of participants was wholly preserved.

### 2.6. Statistical Analysis

To compare biochemical markers with ABO blood group and Rhesus type statistical analysis was performed using SPSS tool version 22.0. Descriptive statistics presented as mean ± standard deviation was performed. Additionally, inferential statistical analysis (*t*-test) was used for investigating the possible differences between two blood groups regarding biochemical markers’ status. One-way ANOVA test further evaluated possible differences in lipidemic and anemia predisposition among blood groups. In all statistical analyses, the level of significance (*p*-value) was set at α = 0.1. The independent variables with *p* < 0.1 in bivariate correlation analysis were enrolled in binary logistic regression for further analysis. A logistic regression model was used to examine the effect of particular independent variables on different ABO blood groups.

## 3. Results

### 3.1. Characteristics of the Study Population

In Table 1 and Table 2, the sociodemographic characteristics such as age-sex matched, BMI, and smoking habits of all participants divided into the different ABO blood groups and Rhesus blood groups, respectively, are shown. This study incorporated about 70% of the healthy regular blood donors’ pool, our population is considered representative of the Naousas Blood Bank. Blood group A comprised of 55 individuals, while B, O, and AB of 32, 51, and 12, respectively. In the whole series of 150 rBD, 132 (88%) were found RhD positive and 18 RhD negative (12%). The most prevalent blood group was A (36.6%) (60% male, 40% female), followed by group O (34%), (50% male, 50% female), and group B (45% male, 55% female) (21.3%). The least common blood group was AB (50% male, 50% female). The prevalence of Rh positive and negative distribution in the studied population was 88% (55% male, 45% female) and 12% (53% male, 47% female), respectively. BMI analysis (range 26.3–26.8) presented that the evaluated groups were all overweight without presenting any significant difference in all blood groups. Data from Eurostat confirms that in 2019 in Europe, the age of 40–50 years 58% are overweight, and especially in Greece, 68% of males and 52% of females are overweight in the age 40–50 years (Data from Eurostat). On the other hand, smoking status was assessed and revealed that ABO group O appeared to be the fewer smokers, only 11% in comparison to ABO group A with 28%. Regarding the RHD groups, the absence of protein D revealed less probability of smoking habit with 13% in comparison to 24% of RHD+. The majority of the participants in the study were non-smokers.

### 3.2. Correlations of Biochemical and Hematological Parameters in Different ABO Blood Groups

Considering the small number of individuals in our study (*n* = 150), firstly processing the descriptive statistical analysis, we are taking into consideration only the mean and standard deviation values. Biochemical and hematological parameters related to ABO blood groups are shown in Table 3 and Supplementary Table S1. Analyzing the lipid profile of the blood donors, different blood groups revealed different biochemical character. More particular, in blood group O, higher levels of triglycerides, LDL, and total cholesterol were observed in comparison to the other groups (*p*-value < 0.05). BDs with O blood group presented the highest atheromatic index with 4.46 in comparison with 4.01 (group A) and 3.88 (group B) (*p*-value < 0.05). Interestingly, uric acid and glucose levels of blood group O presented higher amounts in comparison to the other groups (*p*-value < 0.05). In relation to antioxidant status, slight differences have been observed for TAS levels, only blood group AB presented lower levels of TAS in comparison to the other groups. The ALT and AST liver enzymes had no differences between the blood groups as all the participants were healthy BDs and all the biomarkers were between the normal ranges. Furthermore, analyzing the hematological-related parameters, blood group B revealed slightly higher levels of hematocrit and hemoglobin but with no statistical significance, and group AB had significantly higher levels of iron. Looking at the mean concentration of serum ferritin, a difference has been observed in blood group O in comparison to the other groups. (O: 158, A: 107 and B: 70, AB: 10,032 (ng/mL), respectively. In addition to our analysis, further statistical evaluation was employed. Taking into consideration the limitation regarding the sample size in addition to the non harmonized group size among different ABO blood groups, we calculate the mean values between them in order to depict the relationship between blood groups and atheromatic index variables. Two distinct subgroups were observed. Comparing the mean values of the atheromatic index variable for groups A and B vs. AB and O, we observed that were different. Replacing the variables’ values with 1 (for normal) and 0 (for pathological), we observed that the major differentiations are revealed regarding total cholesterol, triglycerides, and LDL values. (Supplementary Table S1). Comparing the binary values (normal vs. non normal) applying the Chi-Square test, we observed that statistically significant differences appeared between Atheromatic index and glucose variables (*p* = 0.054 and *p* = 0.039 < 0.1, respectively) among ABO blood group groups. Considering that only atheromatic index variables are linearly correlated with ABO blood group groups, the atheromatic index was used in the logistic regression model to determine the effect of the ABO blood group on the atheromatic index appearance. In the binary logistic regression analysis, the model with gender, age, BMI, and blood group was apparently correlated with atheromatic index values. The Hosmer and Lemeshow test with *p* values of 0.72, effectively indicated the better model goodness of fit and further highlighted the role of the blood group A in the model, compared with blood group O (Supplementary Table S2).

### 3.3. Correlations of Biochemical and Hematological Parameters in Different Rh-Blood Groups

Biochemical and hematological parameters related to Rhesus types are shown in Table 4. Considering the lipid profile and hematological-related parameters, the same trend of blood group O has been observed for the positive-Rhesus type in compare with the negative one (AI: Rh+ 4.21, vs. Rh− 3.81, Ferritin levels: Rh+ 125.5 ng/mL vs. Rh− 113.2 ng/mL (*p*-values 0.05) Iron: Rh+ 110.24 μg/dL vs. Rh− 102.12 μg/dL, Ht%: Rh+ 45.2 vs. Rh− 43.4, Hb/g/dL: Rh+ 15.85 vs. Rh− 14.24, Glucose levels: Rh+ 104.5 mg/dL vs. Rh− 97.5 mg/dL). In relation to antioxidant status, the positive-Rhesus type presented significantly higher levels of antioxidants (TAS: Rhesus+ 258.35 mM vs. 241.5 mM Rhesus –in comparison to the negative-Rhesus type.

### 3.4. Correlations of Biochemical and Hematological Parameters according to Gender in Different Blood Groups

The biochemical and hematological characteristics related to gender and ABO blood group are shown in Table 5.

There was a gender dependent association between lipid profile, hyperlipidemia risk, and ABO blood group. Higher triglycerides levels were observed in male blood group B and lower triglycerides levels in female blood group AB, although in the same female group AB was noted a higher hyperlipidemia risk (LDL, total cholesterol levels, and Atheromatic index were higher than in the other groups and HDL levels lower). On the other hand, the statistically significantly higher atheromatic index (5.3, *p* < 0.1, Table 5) in males was noted in blood group B (*p*-value < 0.05). Only total cholesterol levels were higher in males with blood group A. Higher Total antioxidant levels (258.12 mM) in males with blood group O and lower in males with blood group AB (189.25 mM) (*p*-value <0.05) compared to groups A (242.35 mM) and B (242.85 mM) a significant differentiation among the blood groups neither the ABO nor the Rhesus type. It is well known that serum iron, serum ferritin, uric acid, Ht, and Hb are sex related parameters. Uric acid was found to increase in group B in females (5.38 mg/dL) compared to the other blood groups and in group O in males (6.45 mg/dL). In men, serum ferritin levels were increased in blood group O (*p*-value < 0.05) and in females in blood group B. As expected in all-female groups, ferritin levels were lower than in males (Table 5). Hematological parameters Ht and Hb were not associated with gender and blood group. There was no evidence for a relationship between the different blood groups and blood Glucose levels in the two genders. The binary logistic regression analysis revealed a strong statistically significant association of the atheromatic index with gender (Supplementary Table S2).

### 3.5. Limitations of the Study

Certain limitations of this study should also be suppressed. First, the sample size of this study was small (*n* = 150), which resulted in small numbers in some subgroups, particularly the relatively small number of donors with blood group AB (*n* = 12). Lack of associations may therefore reflect limited statistical power to detect smaller (but potentially important) differences between groups. This also limits the generalizability of study findings. However, because this study incorporated about 70% of the healthy blood donors’ pool, our population is considered representative of the Naousas Blood bank. Secondly, other factors such as age, smoking habits, and BMI also impacted our study.

The findings of this study are, however, limited to a single blood bank, and therefore the results may not be generalizable to other blood banks/blood collection agencies. Finally, the study population was ethnically very homogenous as all participants except two were ethnic Greeks, so the findings may only be generalizable to the majority population.

## 4. Discussion

Various systemic diseases, including cardiovascular diseases, diabetes, cancer, and COVID-19, are known to be immune inflammatory responses and are associated with oxidative stress and pathological biomarkers. The rapid spread of COVID-19 presented associations between ABO and Rh blood groups with COVID-19 and further evidence for associations between A and O blood groups too [31]. The role of ABO antigens in the pathogenesis of different diseases is already attempted to be investigated [16,32,33,34]. The frequency of blood groups in our study appears different from the general Greek population. Kremastinou et al. clearly demonstrated that antigens identifying blood group B present a higher distribution in North Greece (Macedonia) [35] as in our study too, while the remaining blood groups are equally distributed within the Greek population and parts of the country.

A meta-analysis study support that the non O blοod group is a risk factor for thrombosis and arterial infraction [36,37]. Higher Low Density Lipoprotein levels in non O blood groups confirm these findings [38,39]. In a pilot study among the sub-Africa population, Ischemic Disease risk was higher in the A blood group than in the other groups. There is not enough evidence why the ABO system is a risk factor for cardiovascular disease. Blood groups A and B compared to O were not significantly associated with cardiovascular risk factors although the AB blood group showed a higher risk for stroke than in O blood groups. Factor VIII was significantly lower in the O blood group population [13]. Also, the A1 blood group was associated with myocardial infarction risk [40]. The reason for the relationship between different blood groups and cardiovascular disease risk is largely unknown. Different haplotypes are common in different races and this is a fact that can explain differences between studies about the association of blood groups with cardiovascular disease risk factors [13]. In conclusion, individuals with group O are at lower risk for thromboembolism because they have lower plasma FVIII and vWF levels, but are at higher risk of bleeding than group A individuals. Due to higher levels of FVIII in blood group A, this group is at higher risk of ischemic heart disease and myocardial infarctions. Coronary atherosclerosis is 44% lower for blood group O and group ex has a high risk of stroke compared to group O [40,41]. In an investigation about hyperlipidemia was reported that triglycerides, LDL, and total cholesterol levels were higher in A, B, and AB blood groups, and the HDL levels were lower [42]. In our study, this investigation is confirmed. Non O blood group and negative rhesus could play a protective role against atheromatic plaque formation. The female AB group have a higher hyperlipidemia risk than other female blood groups, and the B blood group seems to be a risk for hyperlipidemia in male.

The O Blood group population had a lower risk, while the B group higher risk of diabetes mellitus type 2 but no association with the Rhesus blood group was found in a large study in France. B+ individuals had the highest risk than AB+ followed by A− and A+ [43]. In other studies, the results were controversial where AB groups had a protective effect and A blood group had higher blood sugar levels [42]. In Iraq, researchers found higher total cholesterol and higher blood glucose in blood group O individuals, and in a large study in Bangladesh, no association between ABO blood groups and T2DM was reported too. In our study, we found no significant difference between ABO blood groups, and serum glucose levels but higher glucose serum levels in positive Rhesus against negative. According to oxidative stress in the different blood groups there is not much evidence. In a study in 2016, there were no differences in the antioxidant enzymes between the different blood groups but there was found higher levels of superoxides and lower levels of MDA in group O than in the other groups [44]. This can be confirmed by our data that the non O group and RHD + have significantly higher levels of antioxidants than in other blood groups. The lower antioxidant status was evaluated in the AB blood group. Anemia is associated with decreased ferrum and ferritin levels, as well as hemoglobin and hematocrit, and is the main factor for blood donation [45]. Increased ferritin levels are found in individuals with inflammation and insulin resistance [46]. In this study, we investigated the association of these markers with gender and blood groups. A significant association between higher ferritin serum levels and blood group O is observed. This association is gender dependent while it applies to the male group. Higher ferritin levels are observed in female blood group B. Lower ferritin levels in females than in males are observed in our study. This observation can be proved by other studies which suggest that testosterone increases ferritin levels because of inhibiting hepcidin in males [47,48]. In contrast, other studies showed inversed association of ferritin and testosterone but only in normal weight individuals [49,50]. In previous studies, anemia was associated with blood group A and AB and with Rhesus D Positive. The etiology of this association is not determined [51]. In our study, serum iron is higher in the female AB blood group and significantly higher in RhD+. Total antioxidant status is positively correlated with uric acid levels and negatively correlated with lipid peroxidation and ferritin levels. Thus total antioxidant status is multi factor dependent [52,53].

Moreover, a statistically significant observation was that in non O blood groups TAS levels were higher compared to other blood groups, especially for O blood groups with the higher lipid peroxidation. The same was observed in the female group AB (higher atheromatic index, lower TAS levels). The classification of different blood groups is defined by different carbohydrate structures and thus different biochemical profiles seem to associate with different blood groups. Also, race, gender, and ethnicity affect the blood group and lipid profile of individuals. These differences in metabotypes of blood groups can influence health and may contribute to better nutritional and drug therapies [54,55]. Following the same approach, the RHD blood group comparison revealed the complex biochemical pathways behind the few tested associations between the presence and absence of D protein. The RhD blood group is known to be one of the most complex blood groups but it is the most important blood group after the ABO system because it is the main cause of hemolytic disease. D antigen as the most significant and with high immunogenity creates difficulties in understanding the properties of D protein when compared with health markers. Recently, another research group expanded previous knowledge on other associations such as pregnancy-induced hypertension RhD positive, as compared to negative [56]. Also, in our study appeared that negative rhesus could play a protective role against atheromatic plaque formation. Our findings could be combined with previously known associations for RHD and ABO systems correlations and investigate new interesting relations to generate strong further support for the role of ABO and RHD system in our health and protection against a variety of diseases.

## 5. Conclusions

Considering that the atheromatic index variable is linearly correlated with ABO blood groups, the atheromatic index effectively indicated the role of the blood group A in the model, compared with blood group O. Moreover, the O blood group appeared better iron absorption and increased risk for hyperlipidemia and oxidative stress. RHD− group presented also a better lipid profile in comparison to the RHD+ group. With further studies, it can be confirmed that blood groups and types can be considered as risk factors for different diseases alone or in combination, such as CVD, diabetes mellitus, cancer, Covid-19, and the risk profile can be estimated in relation to the other biochemical, hematological parameters and the antioxidant status too.

## Figures and Tables

**Table 1:** Baseline characteristics of all study participants (*n* = 150) according to ABO blood system.

	Blood Group				
	A (*n* = 55) Mean (±SD)	B (*n* = 32) Mean (±SD)	O (*n* = 51) Mean (±SD)	AB (*n* = 12) Mean (±SD)	Non O (*n* = 99) Mean (±SD)
Sex, Male (%)	60	45	50	50	55
Age	42 (±12.08)	43 (±9.55)	46 (±9.26)	44 (±8.54)	42.5 (±10.45)
BMI	26.8 (±2.51)	26.3 (±2.15)	26.4 (±1.87)	26.2 (±1.90)	26.6 (±1.85)
Smoking, Yes (%)	28	19	11	20	23

BMI normal range classified as underweight (BMI: <18.5 kg/m^2^), normal (BMI: 18.5–24.9 kg/m^2^), overweight (BMI: 25–29.9 kg/m^2^), and obese (BMI: ≥30 kg/m^2^). In another classification BMI 25–26.5 kg/m^2^ indicates approximately overweight.

**Table 2:** Baseline characteristics of all study participants (*n* = 150) according to Rhesus type.

	Rhesus Type	
	RhD+ (*n* = 132) Mean ± SD	RhD (*n* = 18) Mean ± SD
Sex, Male (%)	48	55
Age	42.3 (± 11.07)	43.2 (± 9.18)
BMI	26.9 (± 2.12)	25.9 (± 2.14)
Smoking, Yes (%)	24	13

**Table 3:** Biochemical and hematological parameters related to ABO blood system.

	Blood Group Mean (SD +/−)					Statistical Significant *p*-Value
Biochemical Parameters	A	B	O	AB	Non O	
Triglycerides (mg/dL)	105.45 (+34.08)	116.14 (±38.21)	129.65 * (+29.54)	92.25 (+19.65)	119.12 (+23.58)	O-A,B,AB 0.05 0.03 0.02
HDL (mg/dL)	53.24 (+13.21)	56.14 (+11.54)	50.45 (+12.58)	48.13 (+9.54)	56.86 (+13.45)	ns
LDL (mg/dL)	121.42 (+26.05)	126.45 (+24.35)	131.14 * (+29.54)	123.01 (+23.65)	122.32 (+22.55)	0.04 0.02 0.01
Total Cholesterol (mg/dL)	184.52 (±52.65)	200.36 (+49.56)	229.26 * (+65.85)	210.45 (+64.88)	212.24 (+55.85)	0.05 0.02 0.02
Iron (μg/dL)	101.12 (±36.34)	108.35 (±43.48)	108.23 (±39.56)	119.14 * (35.65)	101.12 (41.54)	AB-A,B,O 0.05 0.05 0.05
Ferritin (ng/mL)	107.15 (+54.85)	70.35 (+29.54)	158.45 * (+48.54)	100.32 (+32.45)	85.12 (+49.85)	0.02 0.01 0.01
Uric acid (mg/dL)	5.01 (+1.44)	5.26 (+1.23)	6.03 * (+1.65)	5.25 (+1.34)	5.13 (+1.38)	0.01 0.02 0.05
Atheromatic index	3.88 (±0.94)	4.01 (±0.85)	4.42 * (±0.98)	4.11 (±0.98)	3.95 (±0.96)	0.02 0.03 0.05
AST (U/L)	16.01 (+2.43)	17.12 (+2.87)	18.14 (+2.65)	17.13 (+2.54)	17.74 (+2.61)	ns
ALT (U/L)	19.14 (+3.65)	18.32 (+3.45)	20.45 (+3.44)	18.78 (+3.65)	19.65 (+3.52)	ns
Glucose (mg/dL)	96.47 (+13.54)	98.41 (+13.12)	100.01 * (+12.54)	103.65 (+10.54)	98.31 (+8.65)	0.05 0.05 0.05
TAS (μM)	244.14 (+28.65)	240.71 (+32.54)	239.25 (+31.45)	219.14 * (+29.45)	245.45 (+30.14)	AB-A,B,O 0.05 0.05 0.05
**Hematological** **Parameters**	**A**	**B**	**O**	**AB**	**Non O**	
Ht (%)	44.12 (+3.8)	45.14 (+2.9)	45.01 (+3.45)	43.33 (+2.85)	45.03 (+2.97)	ns
Hb (g/dL)	15.02 (+1.2)	15.05 (+1.05)	15.32 (+1.26)	14.22 (+1.45)	15.18 (+1.12)	ns

Normal ranges: Triglycerides: <150 mg/dL, HDL: 45–100 mg/dL, LDL: <130 mg/dL, Total Cholesterol: <200 mg/dL, Iron: 33–193 μg/dL, Ferritin: 15–150 ng/mL, Uric acid: 2.4–5.7 mg/dL, Atheromatic index (total cholesterol/HDL cholesterol): <4.5. AST: 8–48 U/L, ALT: 7–55 U/L, Glucose: 74–114 mg/dL, TAS: 100–300 μM; Hematological parameters Ht: 37–47%, Hb: 11.5–16,5 g/dL. * Statistical significant *p*-value indicating when needed the different comparing groups.; ns: non-significant.

**Table 4:** Biochemical and hematological parameters related to Rhesus system.

	Rhesus Type Mean (SD +/−)		
Biochemical Parameters	RhD+	RhD−	Statistical Significant *p*-Value
Triglycerides (mg/dL)	128.12 * (±54.85)	116.47 (±48.61)	0.05
HDL (mg/dL)	53.05 (±12.54)	54.47 (±11.54)	ns
LDL (mg/dL)	125.88 * (±27.85)	118.33 (±23.47)	0.05
Total Cholesterol (mg/dL)	234.84 * (±54.57)	201.67 (±45.95)	0.05
Iron (μg/dL)	110.24 (±42.74)	102.12 (±41.85)	ns
Ferritin (ng/mL)	125.5 * (±45.47)	113.2 (±54.74)	0.05
Uric acid (mg/dL)	5.52 (±1.41)	5.35 (±1.08)	ns
Atheromatic index	4.21 (±0.85)	3.81 (±0.73)	ns
AST (U/L)	17.05 (±2.45)	17.24 (±2.12)	ns
ALT (U/L)	19.14 (±3.02)	19.19 (±2.98)	ns
Glucose (mg/dL)	104.5 (±32.45)	97.5 (±29.45)	ns
TAS (m M)	258.35 (±35.45)	241.5 (±39.45)	ns
**Hematological Parameters**	**+**	**−**	
Ht (%)	45.2 (±3.14)	43.4 (±3.25)	ns
Hb (g/dL)	15.85 (±1.45)	14.24 (±1.23)	ns

Normal ranges: Triglycerides: <150 mg/dL, HDL: 45–100 mg/dL, LDL: <130 mg/dL, Total Cholesterol: <200 mg/dL, Iron: 33–193 μg/dL, Ferritin: 15–150 ng/mL, Uric acid: 2.4–5.7 mg/dL, Atheromatic index (total cholesterol/HDL cholesterol): <4.5. AST: 8–48 U/L, ALT: 7–55 U/L, Glucose: 74–114 mg/dL, TAS: 100–300 μM; Hematological parameters Ht: 37–47%, Hb: 11.5–16,5 g/dL. * Statistical significant *p*-value; ns: non-significant.

**Table 5:** Biochemical and hematological parameters in male and female separately related to ABO blood system.

	Blood Group					
	Male Mean (SD +/−)					
Biochemical Parameters	A	B	O	AB	Non O	Statistical Significant *p*-Value
Triglycerides (mg/dL)	128.54 (±32.75)	209.12 * (±29.65)	128.47 (±34.56)	102.75 (±28.65)	168.24 (±30.05)	B-A,O,AB 0.02 0.02 0.03
HDL (mg/dL)	48.25 (±12.25)	45.35 (±11.56)	49.36 (±10.85)	45.25 (±11.98)	47.51 (±12.56)	ns
LDL (mg/dL)	123.47 (±15.65)	143.58 * (±14.32)	132.58 (±15.45)	118.25 (±16.74)	135.78 (±15.57)	0.05 0.05 0.05
Total Cholesterol (mg/dL)	260.5 * 6 (±58.54)	235.85 (±56.85)	228.78 (±51.65)	200.16 (±54.35)	250.36 (±56.58)	0.05 0.05 0.05
Iron (μg/dL)	111.85 (±40.54)	105.25 (±34.54)	102.36 (±32.54)	105.5 (±37.46)	109.45 (±37.53)	ns
Ferritin (ng/mL)	168.02 * (±58.65)	57.36 (±61.32)	330.25 (±52.85)	145.24 (±55.65)	110.14 (±53.26)	0.05 0.05 0.05
Uric acid(mg/dL)	6.02 (±1.23)	6.05 * (±1.36)	6.48 (±1.65)	5.9 (±1.54)	5.91 (±1.46)	ns 0.05 ns
Atheromatic index	4.1 (±0.85)	5.3 * (±0.96)	4.6 (±0.78)	4.29 (±0.79)	4.7 (±0.98)	0.05 0.03 0.02
Glucose (mg/dL)	93.25 (±14.54)	97.38 (±16.54)	98.48 (±17.65)	94.15 (±16.65)	94.87 (±15.65)	ns
TAS (mM)	242.35 (±21.54)	242.85 * (±22.65)	258.12 (±24.65)	189.25 (±21.36)	243.32 (±22.65)	ns 0.05 0.05
**Hematological Parameters**	**A**	**B**	**O**	**AB**	**Non O**	
Ht (%)	44.74 (±3.22)	44.25 (±3.08)	45.01 (±3.65)	44.55 (±3.12)	44.12 (±3.11)	ns
Hb (g/dL)	15.02 (±1.56)	14.28 (±1.36)	15.01 (±1.47)	14.95 (±1.32)	14.4 (±1.45)	ns
**Female Mean (SD +/−)**						
**Biochemical Parameters**	**A**	**B**	**O**	**AB**	**Non O**	**Statistical** **Significant** ***p*-Value**
Triglycerides (mg/dL)	102.45 (±47.94)	101.32 (±45.85)	105.12 (±44.69)	81.75 * (±44.65)	103.14 (±46.91)	AB-A,O,AB 0.05 0.03 0.02
HDL (mg/dL)	61.74 (±13.54)	58.65 (±11.65)	59.45 (±12.85)	51.24 * (±13.58)	59.15 (±13.3)	0.04 0.05 0.05
LDL (mg/dL)	120.24 (±15.65)	113.45 (±13.85)	117.65 (±12.65)	127.24 * (±14.93)	117.45 (±14.35)	0.05 0.05 0.05
Total Cholesterol (mg/dL)	196.14 (±22.25)	200.21 (±19.65)	226.52 (± 24.98)	220.74 * (±23.65)	200.32 (±22.35)	0.05 0.05 0.05
Iron (μg/dL)	101.12 (±39.65)	105.25 (±37.56)	110.36 (±43.61)	133.54 (±44.78)	104.24 (±41.35)	ns
Ferritin (ng/mL)	40.56 (±53.53)	127.12 * (±51.12)	72.45 (±23.12)	55.31 (±22.11)	88.12 (±35.65)	0.05 0.05 0.05
Uric acid(mg/dL)	4.81 (±1.12)	5.38 * (±1.25)	5.23 (±1.36)	3.94 (±1.19)	4.71 (±1.38)	0.05 0.05 0.05
Atheromatic index	3.51 (±0.89)	3.72 (±0.79)	3.59 (±0.94)	3.94 * (±0.79)	3.72 (±0.84)	0.05 0.05 0.05
Glucose (mg/dL)	96.54 (±12.36)	111.14 (±19.65)	102.54 (±14.65)	108.54 (±16.35)	104.45 (±16.42)	ns
TAS (mM)	237.14 (±27.65)	245.65 * (±29.43)	239.55 (±27.42)	234.84 (±23.35)	242.15 (±25.65)	ns
**Hematological Parameters**	**A**	**B**	**O**	**AB**	**Non O**	
Ht (%)	46.02 (±3.11)	45.21 (±2.65)	45.22 (±2.11)	44.32 (±3.15)	45.36 (±2.95)	ns
Hb (g/dL)	15.12 (±1.32)	15.14 (±1.11)	15.32 (±1.65)	14.85 (±1.02)	15.21 (±1.10)	ns

* Statistical significant *p*-value indicating when needed the different comparing groups; ns: non-significant. Normal ranges: Triglycerides: <150 mg/dL, HDL: 45–100 mg/dL, LDL: <130 mg/dL, Total Cholesterol: <200 mg/dL, Iron: 33–193 μg/dL, Ferritin: 15–150 ng/mL, Uric acid: 2.4–5.7 mg/dL, Atheromatic index (total cholesterol/HDL cholesterol): <4.5. AST: 8–48 U/L, ALT: 7–55 U/L, Glucose: 74–114 mg/dL, TAS: 100–300 μM; Hematological parameters Ht: 37–47%, Hb: 11.5–16.5 g/dL.

## Data Availability

Data is contained within the article.

## Supplementary Materials

The following supporting information can be downloaded at: https://www.mdpi.com/article/10.3390/clinpract12030045/s1, Table S1: Representation of the biochemical values with 1 for normal (green color) and 0 for pathological (yellow color); Table S2: The Hosmer and Lemeshow test representation, Blood group 0 was compared with the other tested blood groups.

## Abbreviations

AI	Atheromatic Index
ALT	Alanine aminotransferase
AST	Aspartate transaminase
B	Blood
CDV	Cardiovascular disease
CH	Cholesterol
Hb	Hemoglobin
Ht	Hematocrit
Fe	Ferritin
FVIII	Coagulation Factor
HDL	High density lipoprotein
LDL	Low density Lipoprotein
U.A	Uric acid
VWF	von Willebrand factor
S.I	serum iron
TAS	Total Antioxidant Status
Tr	Triglycerides
