# Supplementary material for: The Clinical Utility of ABO and RHD Systems as Potential Indicators of Health Status, a Preliminary Study in Greek Population
Source: Clin Pract. 2022 Jun 7;12(3):406–18. doi: 10.3390/clinpract12030045 (PMC9221977; doi:10.3390/clinpract12030045)
Supplement: Supplementary file 1 [file clinpract-12-00045-s001.zip › clinpract-1665332-supplementary.pdf]

Supplemental Tables

Table S1. *Representation of the biochemical values with 1 for normal (green color) and 0 for pathological (yellow color). (See Abbreviations).*

ID	B. group	Rhesus	Ht	Hb	S.I	Fe	U.A	CH	Tr	HDL	LDL	A.I	SGOT	SGPT	Gl	BMI
	O	-														
	O	-														
	O	-														
	O	-														
	O	-														
	O	-														
	O	-														
	O	-														
	O	-														
	O	-														
	O	-														
	O	+														
	O	+														
	O	+														
	O	+														
	O	+														
	O	+														
	O	+														
	O	+														
	O	+														
	O	+														
	O	+														
	O	+														
	O	+														
	O	+														
	O	+														
	O	+														
	O	+														
	O	+														
	O	+														
	O	+														
	O	+														
	O	+														
	O	+														
	O	+														
	O	+														
	O	+														
	O	+														
	O	+														
	O	+														
	O	+														
	O	+														
	O	+														
	O	+														
	O	+														
	O	+														
	O	+														
	O	+														
	O	+														
	O	+														
	A	-														
	A	-														
	A	-														
	A	-														
	A	-														
	A	-														
	A	-														
	A	-														
	A	-														
	A	-														
	A	-														
	A	-														
	A	-														
	A	-														
	A	-														
	A	+														
	A	+														
	A	+														
	A	+														
	A	+														
	A	+														
	A	+														
	A	+														
	A	+														
	A	+														
	A	+														
	A	+														
	A	+														
	A	+														
	A	+														
	A	+														
	A	+														
	A	+														
	A	+														
	A	+														
	A	+														
	A	+														
	A	+														
	A	+														
	A	+														
	A	+														
	A	+														
	A	+														
	A	+														
	A	+														
	A	+														
	A	+														
	A	+														
	A	+														
	A	+														
	A	+														
	A	+														
	A	+														
	A	+														
	A	+														
	AB	+														
	AB	+														
	AB	+														
	AB	+														
	AB	+														
	AB	+														
	AB	+														
	AB	-														
	AB	-														
	AB	-														
	AB	-														
	AB	-														
	B	+														
	B	+														
	B	+														
	B	+														
	B	+														
	B	+														
	B	+														
	B	+														
	B	+														
	B	+														
	B	+														
	B	+														
	B	+														
	B	+														
	B	+														
	B	+														
	B	+														
	B	+														
	B	+														
	B	+														
	B	+														
	B	+														
	B	+														
	B	+														
	B	+														
	B	+														
	B	-														
	B	-														

Table S2. *The Hosmer and Lemeshow test representation, Blood group 0 was compared with the other tested blood groups.*

Hosmer and Lemeshow Test

Step	Chi-square	df	Sig.
3	8,595	4	,072

Variables in the Equation

		B	S.E.	Wald	df	Sig.	Exp(B)
Step 3 [a]	GenderF1M0(1)	,837	,426	3,856	1	,050	2,310
	Blood group *0*			7,728	3	,052	
	Blood group *A*	,898	,436	4,239	1	,040	2,454
	Blood group *B*	-,408	,551	,549	1	,459	,665
	Blood group *AB*	-19,969	18491,146	,000	1	,999	,000
	BMI	-,210	,059	12,698	1	,000	,811
	Constant	3,953	1,425	7,695	1	,006	52,082

a. Variable(s) entered on step 3: GenderF1M0.
